# Seroprevalence and Passive Clinical Surveillance of West Nile Virus in Horses from Ecological High-Risk Areas in Western Romania: Exploratory Findings from a Cross-Sectional Study

**DOI:** 10.3390/microorganisms13081910

**Published:** 2025-08-16

**Authors:** Paula Nistor, Livia Stanga, Andreia Chirila, Vlad Iorgoni, Alexandru Gligor, Alexandru Ciresan, Ionela Popa, Bogdan Florea, Mirela Imre, Vlad Cocioba, Ionica Iancu, Janos Degi, Viorel Herman

**Affiliations:** 1 Department of Infectious Diseases and Preventive Medicine, Faculty of Veterinary Medicine, University of Life Sciences “King Mihai I” from Timişoara, 300645 Timişoara, Romania; paula.nistor@usvt.ro (P.N.); vlad.iorgoni@usvt.ro (V.I.); alexandru.gligor@usvt.ro (A.G.); janosdegi@usvt.ro (J.D.); viorel.herman@fmvt.ro (V.H.); 2 Discipline of Microbiology, Faculty of Medicine, “Victor Babes” University of Medicine and Pharmacy, Eftimie Murgu Square 2, 300041 Timişoara, Romania; 3 Department of Surgery, Faculty of Veterinary Medicine, University of Life Sciences “King Mihai I” from Timişoara, 300645 Timişoara, Romania; chirilaandreia@yahoo.com (A.C.); alexandru.ciresan@usvt.ro (A.C.); 4 Department of Semiology, Faculty of Veterinary Medicine, University of Life Sciences “King Mihai I” from Timişoara, 300645 Timişoara, Romania; ionela.popa@usvt.ro; 5 Department of Internal Medicine, University of Life Sciences “King Mihai I” from Timişoara, 300645 Timişoara, Romania; bogdan-alexandru.florea.fmv@usvt.ro; 6 Department of Parasitology, University of Life Sciences “King Mihai I” from Timişoara, 300645 Timişoara, Romania; mirela.imre@usvt.ro; 7 Department of Animal Husbandry, University of Life Sciences “King Mihai I” from Timişoara, 300645 Timişoara, Romania; vlad-mihai.cocioba.fmv@usvt.ro

**Keywords:** West Nile virus, seroepidemiology, passive clinical surveillance, IgM ELISA, neuroinvasive disease, vector-borne zoonosis

## Abstract

This cross-sectional study evaluated the seroprevalence and clinical impact of West Nile virus (WNV) infection in horses from three ecologically high-risk counties in western Romania (Timiș, Arad, and Bihor) between 2023 and 2025. A total of 306 unvaccinated horses were tested using a commercial ELISA, with 8.17% testing positive for WNV antibodies, indicating prior exposure. Passive surveillance for clinical signs during mosquito seasons identified 16 horses with acute neurological symptoms, four of which were confirmed as clinical cases based on WNV-specific IgM positivity, suggesting probable silent WNV circulation in the region. The overall case fatality rate among confirmed clinical cases was 25.0%. WNV seropositivity was highest in Bihor (8.85%), followed by Arad (8.57%) and Timiș (7.32%). Statistical comparisons using χ^2^ tests and binary logistic regression indicated no significant differences in seroprevalence between counties, sexes, or age groups, consistent with the overlapping 95% confidence intervals. These findings suggest the continued silent circulation of WNV in the region and support the integration of equine surveillance into the One Health framework as a potential tool for early detection and risk mitigation. However, in the absence of molecular confirmation (e.g., RT-PCR or virus isolation), these results should be interpreted as indicative of prior exposure rather than direct evidence of ongoing viral activity.

## 1. Introduction

West Nile virus (WNV) is a mosquito-borne flavivirus of zoonotic concern, widely distributed across Europe, Africa, and the Americas. It circulates primarily between ornithophilic mosquitoes (*Culex* spp.) and birds, with incidental infections occurring in humans and horses, some of which may develop neuroinvasive disease [1].

*Culex* spp. mosquitoes, particularly *Cx. pipiens* and *Cx. modestus*, act as primary vectors of WNV in Europe, acquiring the virus from infected birds. Environmental factors such as wetlands, irrigation, and climate conditions influence the abundance of vectors and shape the seasonal dynamics of WNV transmission [2].

In Romania, the first major outbreak of WNV occurred in 1996, resulting in over 800 confirmed human cases. Since then, seasonal transmission has been reported regularly, especially in southern and southeastern regions but also in the west, including in Timiș, Arad, and Bihor counties. Recent entomological and molecular surveillance studies have provided strong evidence of continuous WNV circulation in local mosquito populations. Between 2017 and 2023, *Cx. pipiens* and *Cx. modestus* mosquitoes collected from urban and peri-urban sites in the Bucharest area tested positive for WNV RNA, confirming ongoing transmission of lineage 2 strains [3]. In parallel, a high-throughput microfluidic PCR screening of mosquito pools from eastern Romania detected WNV lineage 2 and, for the first time in the country, Sindbis virus (SINV) RNA in *Cx. modestus*, highlighting the co-circulation of multiple flaviviruses in endemic regions [4]. Genetic analysis of WNV isolates revealed the co-existence of sub-lineages 2a and 2b in both vectors and clinical samples, suggesting repeated introductions followed by localized evolution. The presence of closely related flaviviruses, such as Usutu virus (USUV) and tick-borne encephalitis virus (TBEV), raises concerns about potential cross-reactivity in serological assays and highlights the importance of confirmatory testing in seroprevalence studies [3,5].

In horses, WNV infection is mostly subclinical; however, neurological signs such as ataxia, cranial nerve deficits, or seizures may occur in a minority of cases, with case fatality rates typically ranging between 25% and 45% in clinically affected individuals [6]. Several Southern and Southeastern European countries, including Serbia, Croatia, Italy, and Hungary, have reported recurrent equine outbreaks of WNV, highlighting regional endemicity. Despite this, equine surveillance remains inconsistent in Romania [7,8,9]. Although WNV vaccines are commercially available, their use remains limited in Romania, and equine vaccination is not systematically implemented.

WNV circulation in Romania has been confirmed both in southern and southeastern regions, such as the Danube Delta, and in western counties including Timiș, Arad, and Bihor. These areas combine favorable ecological conditions such as wetlands, aquaculture systems, and migratory bird flyways [10,11,12]. A recent serological survey also detected WNV antibodies in horses from western Romania and neighboring Bulgaria, suggesting transboundary transmission routes and the need for regional surveillance harmonization [10].

Due to their outdoor exposure and susceptibility to neuroinvasive disease, horses can serve as useful indicators of local WNV activity when included in structured surveillance frameworks. However, it is important to note that IgM-based confirmation, while useful in field conditions, cannot replace molecular diagnostics such as RT-PCR or virus isolation when definitive confirmation is required [13,14].

This study aimed to estimate WNV seroprevalence and identify clinical neuroinvasive cases among unvaccinated horses in three ecologically high-risk counties in western Romania. Through serological testing and passive clinical surveillance, we sought to explore the local burden of infection and assess the potential role of horses in early detection strategies.

## 2. Materials and Methods

### 2.1. Study Area and Design

This cross-sectional observational study was conducted between June 2023 and March 2025 in three western Romanian counties: Timiș, Arad, and Bihor, previously identified as high-risk areas for WNV transmission based on ecological and serological evidence [10]. Although data collection spanned two consecutive mosquito transmission seasons, each horse was serologically sampled only once. Therefore, the study maintained a cross-sectional design at the individual level, with the extended timeframe allowing for broader spatial coverage and logistical feasibility.

Given that horses were enrolled at different time points across two distinct vector seasons, the study may be more accurately described as a repeated cross-sectional survey at the population level. This design introduces the potential for temporal variation in exposure risk, particularly due to annual differences in vector activity and environmental conditions. To minimize such variation, eligibility was restricted to unvaccinated, permanently outdoor-housed horses with at least 12 months of continuous residence in the study area, and uniform sampling procedures were applied across all counties.

Despite these efforts, interannual ecological fluctuations could have influenced exposure levels and should be considered when interpreting seroprevalence estimates. Nonetheless, the individual-level single-timepoint sampling strategy supports the internal validity of the findings by reducing recall and temporal biases.

### 2.2. Study Population and Inclusion Criteria

Horses were selected via purposive sampling based on ecological exposure, specifically, permanent outdoor housing and proximity to mosquito-prone environments such as wetlands, riverbanks, and irrigation systems [2,10]. This non-random approach is considered epidemiologically appropriate in high-risk settings and exploratory contexts [15,16]. However, this ecological selection criterion may have excluded horses from indoor-managed facilities, where viral circulation could occur undetected, potentially underestimating overall exposure.

All horses were aged ≥1 year, continuously resident in the same county for at least 12 months, and unvaccinated against WNV, as confirmed by owner reporting and veterinary records [17,18]. Although efforts were made to exclude vaccinated horses based on veterinary records and owner declarations, no serological differentiation was conducted between vaccine-induced and infection-induced antibodies. Given the limitations of the competitive ELISA in this regard, the possibility of misclassification cannot be entirely excluded. This represents a potential classification bias, as competitive or blocking ELISAs, such as the one used in this study, cannot reliably distinguish between IgG produced by vaccination and that resulting from natural infection, especially in the absence of pre-vaccination baseline data [19]. Therefore, although efforts were made to exclude vaccinated animals, the presence of undetected vaccination cannot be fully excluded and may influence the interpretation of seroprevalence.

This purposive selection strategy, while pragmatic and ecologically relevant, may introduce selection bias and limit the generalizability of results to the broader equine population in the region. The study population included both sexes and a variety of breeds, without stratification, reflecting the typical demographic structure of the regional equine population. The body condition score (BCS) was assessed by trained veterinarians using the standardized 9-point Henneke scale [20] at the time of blood sample collection. A detailed summary of the age distribution, sex ratio, and BCS ranges, along with the relative proportion of each age group, is provided in Table 1.

### 2.3. Sample Collection and Handling

Blood was collected once from each horse by jugular venipuncture into sterile vacutainer tubes without anticoagulant. Samples were kept at 4–8 °C during transport, centrifuged at 1500× *g* for 10 min within 12 h, and sera were aliquoted and stored at −20 °C. For long term preservation, sera were transferred to −80 °C prior to testing. All procedures followed the biosafety and specimen handling recommendations for arboviral surveillance provided by the World Organisation for Animal Health (WOAH) [21].

### 2.4. Serological Testing for WNV Antibodies

Two different ELISA kits, ID Screen^®^ West Nile Competition ELISA (IDVet, Grabels, France), INgezim^®^ West Nile IgM ELISA (Ingenasa, Madrid, Spain), were employed in this study for distinct diagnostic purposes. All 306 serum samples were tested in single runs using the ID Screen^®^ West Nile Competition ELISA (IDVet, Grabels, France), following the manufacturer’s instructions. Repeat testing was not performed for all samples, and thus systematic intra plate error cannot be fully excluded.

This assay is validated for equine use, with reported sensitivity of >95% and specificity of >98%, and is recommended by the World Organisation for Animal Health for WNV serological surveillance in horses [22,23,24]. Its analytical performance has been independently confirmed in comparative studies of serological assays for WNV antibodies in equids [14]. Internal positive and negative controls were included on each microplate. The optical density (OD) was measured at 450 nm using a Sunrise™ microplate reader (Tecan GmbH, Grödig, Austria). The results were interpreted based on the inhibition percentage (IP): IP ≥ 40% = positive; IP ≤ 30% = negative; IP 30–40% = equivocal. Samples yielding equivocal results (IP 30–40%) were re-tested in duplicate, and all produced definitive results upon repetition.

The INgezim^®^ West Nile IgM ELISA (Ingenasa, Madrid, Spain) was used exclusively for horses presenting clinical signs compatible with acute WNV infection. This assay detects WNV-specific IgM antibodies and demonstrates low cross-reactivity with other flaviviruses such as Usutu virus and tick-borne encephalitis virus [24]. Its diagnostic specificity in equines has been further supported in recent field-based epidemiological studies [25]. Out of the 306 horses tested serologically, 25 (8.17%) were seropositive based on the blocking ELISA. Among these, four horses also met the clinical case definition and were confirmed via IgM ELISA. The remaining 12 horses with compatible clinical signs either tested negative for IgM antibodies or could not be sampled due to owner refusal or late-stage presentation.

### 2.5. Clinical Surveillance and Case Confirmation

Passive clinical surveillance for WNND was conducted during the mosquito seasons (May–October) of 2023 and 2024. Local equine veterinarians were sensitized to recognize hallmark neurological signs, including ataxia, cranial nerve dysfunction, muscle fasciculations, behavioral changes, recumbency, and seizures, in accordance with established clinical guidelines [26]. Suspected WNND cases were reported using a standardized notification form and referred to the coordinating research team. All cases were clinically assessed by a core group of experienced equine veterinarians using a unified checklist to minimize interobserver variability. Blood samples were collected within five days of symptom onset and tested for WNV-specific IgM antibodies. A clinical case was considered confirmed when acute neurological signs coincided with IgM positivity, in line with accepted field criteria for probable equine WNND [26].

A total of four cases met the clinical case definition. These represented the only horses confirmed within the defined surveillance period. No cerebrospinal fluid or post-mortem samples were available for RT-PCR confirmation, and no ancillary testing for differential diagnoses (e.g., EHV-1, rabies) was feasible due to infrastructural constraints. Confirmed cases were geographically distributed as follows: two in Timiș County (including the only recorded fatality), one in Arad, and one in Bihor. The number of veterinarians involved and the total count of untested or IgM-negative suspect cases could not be reliably recorded. Consequently, milder or atypical WNND presentations may have remained undetected, and the actual clinical burden in the region is likely underestimated. Because case capture in this study relied on voluntary practitioner reports, implementing active sentinel surveillance, through systematic monitoring of selected equine populations, could improve case detection, including subclinical and atypical presentations, and provide more robust epidemiological data. Given the passive nature of the surveillance system and its reliance on voluntary reporting, these limitations are expected. Implementing active sentinel surveillance programs may improve the detection of both typical and subclinical WNV cases in equine populations.

### 2.6. Data Management and Statistical Analysis

The data were anonymized and managed in a centralized Microsoft Excel^®^ 365 database. Quality control was ensured via double-entry validation and internal consistency checks [27]. Descriptive statistics were used to summarize WNV seroprevalence stratified by county, sex (male/female), and age group (1–5 years, >5 years). Binomial 95% confidence intervals (CI) for proportions were calculated using the Wilson score method, appropriate for small or skewed sample sizes [28]. The clinical case fatality rate and its 95% CI were also calculated using the Wilson method to account for uncertainty due to the low number of confirmed cases. All analyses were conducted using Epi Info™ version 7.2 (CDC, Atlanta, GA, USA). To evaluate potential associations between seropositivity and demographic or geographic variables, χ^2^ tests of independence and binary logistic regression were performed. The variables included in the logistic regression model were county, sex, and age group. All analyses were conducted using Epi Info™ version 7.2 (CDC, USA). Given the exploratory nature of the study, the results of these inferential tests are presented descriptively and should be interpreted with caution. Descriptive spatial analysis was performed using QGIS version 3.28 (QGIS.org), mapping seropositive cases and clinical WNND reports at the county level relative to known mosquito habitats (e.g., wetlands, rivers). Due to the unavailability of exact geolocation data and a limited sample size, no inferential spatial models (e.g., kriging, cluster detection) were applied. Consequently, spatial patterns were interpreted descriptively, in accordance with current best practices in ecological epidemiology [28,29].

Due to the absence of precise point-level geographic coordinates and confidentiality restrictions agreed upon with horse owners and veterinary practitioners, mapping was performed exclusively at the county level. Consequently, spatial cluster analyses could not be conducted, and the maps presented should be interpreted as purely descriptive.

## 3. Results

### 3.1. Serological Findings

**Figure 1:**
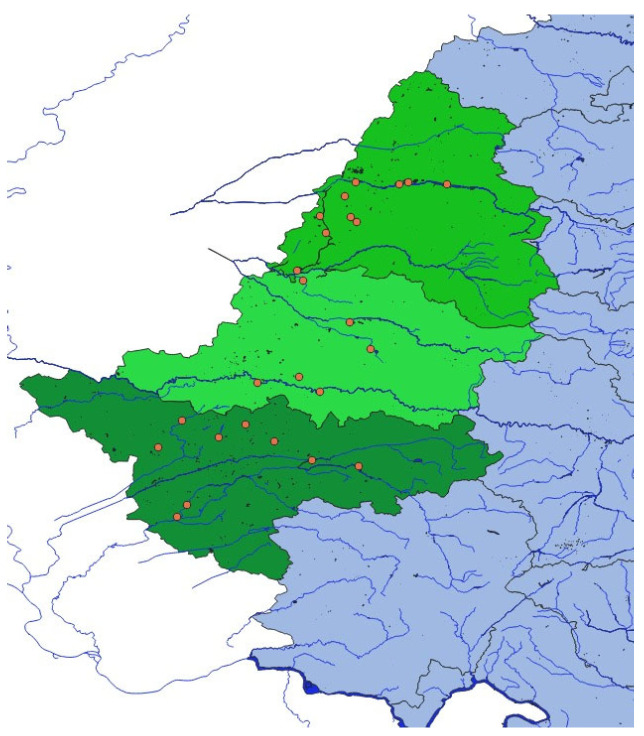
Geographic distribution of WNV-positive horses in the study area of western Romania. Red dots indicate the locations of positive cases detected during the survey.

A total of 306 horses from three counties in western Romania were tested for the presence of WNV-specific antibodies using a commercial epitope-blocking ELISA. Of these, 25 (8.17%) horses tested seropositive. County-specific seroprevalence was as follows: Bihor 8.85%, Timiș—7.32%, and Arad—8.57%, with wide 95% confidence intervals due to limited sample sizes (Table 2, Figure 1).

The highest seroprevalence was observed in Bihor County (8.85%), which borders Hajdú-Bihar County in Hungary, an area previously reported to have elevated WNV activity [30]. These serological data suggest a possible localized circulation of WNV in equine populations within these ecologically vulnerable zones, consistent with findings from other Central and Eastern European regions, such as Hungary (26%) [17] and Serbia (12%) [31]. This interpretation remains provisional in the absence of molecular confirmation.

Statistical comparisons using χ^2^ tests and binary logistic regression indicated no significant differences in seroprevalence between counties, sexes, or age groups, consistent with the overlapping 95% confidence intervals. Similarly, no clear pattern of variation was observed in the body condition score. Full stratified data are available upon request.

To evaluate potential associations between seropositivity and demographic or geographic variables, χ^2^ tests of independence and binary logistic regression were performed. The χ^2^ tests showed no statistically significant differences by county (χ^2^ = 0.35, *p* = 0.84), sex (χ^2^ = 0.09, *p* = 0.77), or age group (χ^2^ = 0.86, *p* = 0.65). In the binary logistic regression model, none of the predictors were significantly associated with seropositivity: county (OR range: 0.81–1.23, *p* > 0.05), sex (OR = 1.07, 95% CI: 0.46–2.48, *p* = 0.87), and age group (OR = 1.15, 95% CI: 0.49–2.69, *p* = 0.75). These findings indicate no detectable effects of age, sex, or county within the dataset analyzed.

Based on the reported diagnostic performance of the ID Screen^®^ West Nile Competition ELISA (sensitivity = 95%, specificity = 98%), the apparent seroprevalence of 8.17% (25/306) corresponds to an estimated true prevalence of 6.63%, calculated using the Rogan–Gladen formula. The corresponding positive predictive value (PPV) was 80.8%, and the negative predictive value (NPV) was 99.6%, assuming constant test characteristics across the study population. These estimates highlight the high reliability of negative results but indicate that, in the absence of molecular confirmation, a proportion of positive results may represent false positives.

### 3.2. Clinical WNND Cases and Outcomes

During the 2023 and 2024 mosquito seasons (May–October), 16 horses were reported with acute neurological signs compatible with WNND. Of these, four tested positive for WNV-specific IgM antibodies, fulfilling field-based criteria for probable WNND [10,11]. All confirmed cases occurred between late July and early October, coinciding with peak Culex spp. activity [6]. The remaining 12 cases either tested negative or could not be sampled due to owner refusal or late presentation beyond the recommended diagnostic window, representing a substantial limitation in attributing causality.

The observed neurological signs among the four confirmed cases included ataxia (*n* = 3), muscle fasciculations or tremors (*n* = 2), cranial nerve deficits (facial droop and dysphagia; *n* = 1), behavioral changes (e.g., hyperexcitability or depression; *n* = 1), and severe signs such as recumbency and limb paresis (*n* = 1). Seizures were not observed. Due to the small number of confirmed cases, these observations are descriptive and should not be interpreted as prevalence estimates. Clinical signs typically developed within 5–10 days after presumed exposure, aligning with the known incubation period in horses [26]. These patterns are consistent with previous reports of equine WNND in Europe and North America [8,10,29,31].

One horse from Timiș County was euthanized due to irreversible neurological deterioration, resulting in an overall case fatality rate of 25.0% (1/4; 95% CI: 4.6–69.9%), calculated using the Wilson method for small samples [28]. The other three horses, from Arad, Bihor, and Timiș, fully recovered after supportive therapy, including anti-inflammatories and fluid replacement, in accordance with AAEP guidelines [26]. Recovery times ranged from two to four weeks, and no relapses or persistent neurological deficits were observed during the 4–6 week follow-up period.

A summary of clinical cases, outcomes, and case fatality rates by county is presented in Table 3. Given the passive nature of surveillance, the absence of cerebrospinal fluid or molecular testing (e.g., RT-PCR), and the lack of ancillary diagnostics (e.g., for EHV-1), the true burden of clinical disease is likely underestimated. Future implementation of active sentinel surveillance could improve the detection of both typical and atypical presentations.

### 3.3. Geographic and Environmental Correlation

Descriptive spatial analysis indicated that both seropositive horses and WNND clinical cases were primarily located near wetland habitats, aquaculture areas, and river basins, which are environments conducive to *Culex* mosquito proliferation. This ecological clustering appears to align with broader European reviews that associate such environments with WNV distribution and seasonal resurgence [2,7].

Bihor County exhibited the highest WNV seroprevalence among the surveyed counties (8.85%), followed by Arad (8.57%) and Timiș (7.32%) (Table 2). This pattern may reflect the influence of favorable environmental conditions and cross-border proximity to endemic regions in Hungary [30]. The highest number of WNND clinical cases (*n* = 2) and the only reported fatality were recorded in Timiș, while Arad showed moderate seropositivity with no associated mortality.

Geospatial mapping using QGIS 3.28 suggested that 76% (19/25) of seropositive horses were located within 2 km of wetlands, irrigation systems, or riverbanks compared to 58% (163/281) of seronegative horses. Although these differences are ecologically plausible, no formal spatial statistical analysis was performed due to the limited resolution of the geolocation data and the absence of concurrent entomological surveillance. As such, the findings remain exploratory and cannot confirm any causal spatial relationships.

While these observations highlight a potentially important ecological dimension, the absence of high-resolution data and spatial modeling (e.g., cluster detection using SaTScan or risk surface interpolation via kriging) precludes definitive conclusions. Future studies integrating environmental, entomological, and spatial data streams could better elucidate environmental risk predictors and support the development of more robust One Health surveillance frameworks.

## 4. Discussion

In a study from 2023 in Romania, WNV was detected in *Culex pipiens*, *Culex modestus*, and *Aedes vexans*, confirming its continued circulation in eastern and southeastern regions. Phylogenetic analysis revealed WNV lineage 2 (Central-Southeast European clade), indicating a wider distribution than previously known. Additionally, it was reported as the first molecular detection of SINV in Romania, although confirmation by conventional RT-PCR and virus isolation was unsuccessful. These findings emphasize the value of ongoing molecular surveillance amid environmental changes that may influence arbovirus dynamics [4].

In a 2024 Romanian study, WNV lineage 2 was identified in mosquitoes, birds, and humans, demonstrating sustained circulation from 2017 to 2023. While most detections occurred in *Culex pipiens*, *Aedes albopictus* was also implicated, indicating potential changes in vector dynamics. Both sub-lineages 2a and 2b were present, with multiple clusters within 2a, consistent with repeated introduction events followed by local viral evolution. These results highlight the complex epidemiology of WNV in Romania and reinforce the need for ongoing molecular surveillance to track viral diversity and dissemination [3].

The findings of this study suggest the possible silent circulation of WNV among equine populations in western Romania, with a seroprevalence of 8.17%, similar to values reported in ecologically vulnerable areas of eastern Hungary (26%) [17] and northern Serbia (12%) [31] and within the range observed in recent cross-sectional surveys from Bulgaria (3.97%) [10].

However, this interpretation remains provisional given that only a small proportion of neurological cases were IgM-positive and no molecular or post-mortem confirmation was performed. Future investigations should combine serology with RT-PCR and, where feasible, cerebrospinal fluid analysis to strengthen diagnostic certainty and better characterize active transmission. The highest rate was observed in Bihor County (8.85%), which borders Hajdú-Bihar County in Hungary, a known WNV hotspot [8]. This spatial pattern is in line with previous findings indicating that wetlands, fish farms, and irrigated zones support *Culex* spp. mosquito persistence, contributing to epidemiological clustering in Hungary and ecological modeling in Italy and other European countries [30,32,33,34].

Most seropositive horses were asymptomatic, supporting estimates in the literature that 80% of WNV infections in equines are subclinical [35]. However, the identification of four neuroinvasive cases, including one fatality, emphasizes the risk of severe clinical outcomes in unvaccinated populations. The neurological signs observed, including ataxia and cranial nerve deficits, were consistent with previous outbreak descriptions [8,36] and occurred between July and September, coinciding with peak *Culex* spp. activity [33,34,37]. Due to the absence of concurrent entomological or meteorological data, seasonal transmission patterns cannot be fully assessed.

The observed seroprevalence in this study (8.17%) is comparable to previous European investigations, such as the 2020 survey reporting 8.1% in unvaccinated horses from high-risk wetland areas [23]. In contrast, data from Hungary (26%) [17], Serbia (12%) [31], and other European countries, including Italy (up to 38% in outbreak zones) [38], show a wider range, often higher in wetland adjacent areas with documented human and avian cases. The case fatality rate observed here (25.0%) is similar to other reported cases in Europe (15–21%), potentially due to differences in lineage (lineage 2 in Europe), improved clinical management, or underrecognition of cases in rural areas. However, our small clinical sample limits strong conclusions [39,40,41].

Recent Romanian studies further confirm the virus’s ongoing presence and growing ecological complexity. In 2023, WNV was detected in *Culex pipiens*, *Culex modestus*, and *Aedes vexans*, confirming its continued circulation in eastern and southeastern regions. Phylogenetic analysis identified lineage 2 (Central-Southeast European clade), indicating a broader distribution than previously recognized. Additionally, the first molecular detection of SINV in Romania was reported, although confirmation by RT-PCR and virus isolation was unsuccessful. In 2024, WNV lineage 2 was found in mosquitoes, birds, and humans, with detections between 2017 and 2023. While most were in *Culex pipiens*, *Aedes albopictus* was also implicated, suggesting evolving vector dynamics. Both sublineages 2a and 2b were identified, including multiple clusters within 2a, indicative of repeated introductions and localized evolution [3,4]. These findings underscore the need for sustained molecular surveillance amid environmental shifts that influence arbovirus patterns.

Horses may serve as useful sentinels in integrated surveillance systems, provided that timely diagnostic access and active monitoring are ensured [42]. Compared to birds or humans, equines may show early, specific neurological signs, facilitating faster clinical suspicion and targeted diagnostics [8]. The spatial overlap between equine seropositivity and human WNND reports, as documented by ECDC and Romanian authorities, demonstrates the value of integrating veterinary surveillance into early warning systems. Nonetheless, Romania lacks a national equine WNV surveillance program, and veterinary reporting remains limited, unlike more established human systems, highlighting the need for integration [12,43].

Successful One Health models in Spain and France have shown that the inclusion of horses improves outbreak detection and response [23,44]. The economic burden due to veterinary care, euthanasia, and performance loss further supports vaccination, which, despite limited uptake in Romania, remains the most effective preventive tool [45].

Several limitations must be acknowledged. The purposive sampling design introduces selection bias [8], and the absence of data on vaccination status, comorbidities, or vector control exposure limits analytical resolution. Although all horses were reported as unvaccinated, confirmation relied solely on veterinary records and owner declarations, without serological differentiation between vaccine-induced and infection-induced antibodies. This limitation introduces the potential for misclassification bias, as competitive ELISA assays cannot reliably distinguish IgG antibodies derived from vaccination versus natural infection [17]. Such misclassification could result in a slight overestimation of true seroprevalence in our study. Passive surveillance, based on voluntary reporting by local veterinarians, may have led to the underreporting of both subclinical and atypical neuroinvasive presentations. Previous equine WNV studies in Europe have documented that up to 50–70% of mild neuroinvasive cases may go unreported under passive systems [46,47], suggesting that the true incidence in our population may be higher than observed. Molecular diagnostics were not feasible due to logistical constraints and the brief viremia phase in horses; thus, detection relied on IgM and epitope-blocking ELISA [44]. A lack of concurrent entomological and avian surveillance reduces ecological insight. Integrated systems in Germany and Spain combining animal, vector, and human data offer more comprehensive models [46,48].

Potential cross-reactivity with other flaviviruses, particularly USUV, cannot be entirely excluded. Still, the commercial ELISA kits used are considered highly specific [47]. Potential cross-reactivity with other flaviviruses, notably Usutu virus (USUV) and tick-borne encephalitis virus (TBEV), cannot be entirely excluded. While the cELISA kit used here has reported specificity above 98% in equine samples, field conditions in areas of co-circulation may still yield occasional false positive results [17]. A major limitation of this study is the absence of confirmatory virus neutralization testing (VNT), the reference method for verifying WNV-specific antibodies. Although a validated cELISA kit with high sensitivity and specificity was employed, cross-reactivity with other flaviviruses cannot be completely excluded. VNT could not be performed due to restricted access to BSL-3 laboratory facilities and resource constraints; therefore, the serological results should be interpreted with caution. Future surveillance efforts should incorporate VNT (or PRNT) confirmation of all positive and equivocal samples to ensure the specificity of WNV seroprevalence estimates [14]. In addition, the lack of access to high-resolution local meteorological data limits the ability to assess seasonal dynamics, which are strongly influenced by environmental variables such as temperature, rainfall, and humidity [7].

To mitigate WNV impact, vaccination should be prioritized in high-risk areas, as it effectively reduces morbidity and mortality [1]. Clinicians should include WNV in differential diagnoses of neurologic cases during mosquito season, even in the absence of fever [26]. Expanding access to IgM ELISA testing is crucial for timely diagnosis [49]. Educational outreach for horse owners and stable workers should emphasize early symptom recognition, vector control, and vaccination benefits [26]. Integrating equine data into national reporting systems and enhancing cross-sector collaboration would significantly strengthen One Health preparedness [46].

Future research should include longitudinal serology to track exposure trends and antibody waning [50], molecular methods (RT-PCR, sequencing) to identify circulating lineages and reassortment events [47], and entomological work focused on vector dynamics and insecticide resistance [33]. Predictive GIS-based models incorporating climatic and ecological variables can improve risk forecasting [51]. Effective WNV control depends on interdisciplinary coordination, real-time surveillance, and robust One Health collaboration across veterinary, medical, and environmental sectors [52].

## 5. Conclusions

This study supports the hypothesis of silent WNV circulation in equine populations from western Romania. However, in the absence of direct virological confirmation (e.g., RT-PCR or virus isolation), endemic transmission cannot be definitively established. The observed spatial clustering of seropositive and symptomatic horses in proximity to wetlands, riverbanks, and irrigated agricultural zones underscores the potential role of environmental factors in shaping local transmission dynamics.

These findings suggest the potential sentinel value of horses under active and structured surveillance systems, particularly in ecologically high-risk areas. However, given the limited number of confirmed clinical cases, the passive nature of surveillance, and the lack of active diagnostic confirmation, this potential sentinel role remains provisional. Strategic integration of equine data into active, multi-sectoral surveillance frameworks is warranted to improve early warning capacity and One Health preparedness.

To reduce WNV-related risks for both animals and humans, we recommend implementing targeted vaccination campaigns in ecologically high-risk areas, expanding routine serological surveillance, and strengthening coordination among veterinary, public health, and environmental stakeholders.

## Figures and Tables

**Table 1:** Demographic and body condition score (BCS) distribution of sampled horses.

Age Group (Years)	No. of Horses	% of Total	Sex (M/F)	BCS Range
1–4	32	10.5%	14/18	3–6
5–9	98	32.0%	45/53	4–7
10–14	102	33.3%	38/64	3–6
15–19	54	17.6%	21/33	2–5
≥20	20	6.5%	8/12	2–4
Total	306	100.0%	-----	-----

**Table 2:** Distribution of tested horses, number of seropositive cases, WNV seroprevalence, and 95% confidence intervals by county (2023–2024).

County	Horses Tested	Seropositive	Seroprevalence (%)	95% Confidence Interval
Bihor	113	10	8.85%	4.88–15.53%
Timiș	123	9	7.32%	3.90–13.32%
Arad	70	6	8.57%	3.99–17.47%
Total	306	25	8.17%	5.59–11.78%

**Table 3:** Number of clinical WNV cases, recovery outcomes, and case fatality rates by county.

County	Clinical Cases	Recovered	Euthanized	Case Fatality Rate (%)
Timiș	2	1	1	50.0%
Arad	1	1	0	0.0%
Bihor	1	1	0	0.0%
Total	4	3	1	25.0%

## Data Availability

The original contributions presented in this study are included in the article. Further inquiries can be directed to the corresponding authors.

## Abbreviations

The following abbreviations are used in this manuscript:

WNV	West Nile Virus
WNND	West Nile Neuroinvasive Disease
CNS	Central Nervous System
ELISA	Enzyme-Linked Immunosorbent Assay
PCR	Polymerase Chain Reaction
IgM	Immunoglobulin M
USUV	Usutu Virus
